# Modelling and Laboratory Studies on the Adhesion Fatigue Performance for Thin-Film Asphalt and Aggregate System

**DOI:** 10.1155/2014/819083

**Published:** 2014-06-19

**Authors:** Dongsheng Wang, Junyan Yi, Decheng Feng

**Affiliations:** ^1^School of Transportation Science and Engineering, Harbin Institute of Technology, Harbin 150090, China; ^2^School of Chemical Engineering and Technology, Harbin Institute of Technology, Harbin 150090, China

## Abstract

Adhesion between asphalt and aggregate plays an important role in the performance of asphalt mixtures. A low-frequency adhesion fatigue test was proposed in this paper to study the effect of environment on the asphalt-aggregate adhesion system. The stress-based fatigue model had been utilized to describe the fatigue behavior of thin-film asphalt and aggregate system. The factors influencing the adhesion fatigue performance were also investigated. Experiment results show that asphalt has more important effect on the adhesion performance comparing with aggregate. Basalt, which is regarded as hydrophobic aggregates with low silica content, has better adhesion performance to asphalt binder when compared with granite. The effects of aging on the adhesion fatigue performance are different for PG64-22 and rubber asphalt. Long-term aging is found to reduce the adhesion fatigue lives for rubber asphalt and aggregate system, while the effect of long-term aging for aggregate and PG64-22 binder system is positive. Generally the increased stress amplitude and test temperature could induce greater damage and lead to less fatigue lives for adhesion test system.

## 1. Introduction

Moisture damage is one of the important reasons inducing premature failure in asphalt pavement [1–3], which is thought to occur either within the binder (fracture of cohesive bond) or at the binder-aggregate interface (failure of adhesive bond). Among them the interfacial adhesive bond is considered to play a more important role in moisture damage [4]. Therefore researchers had conducted a lot of studies on the interfacial adhesion mechanisms, experimental methods, and evaluation index in the past years. As to the basic adhesion mechanisms, it had been classified as electrostatic forces, chemical bonding, and adhesion due to surface free energy [1, 4–7]. Among them the surface free energy method is most popularly used to investigate the adhesion bond between aggregate and asphalt binder [6–9].

In addition to the surface free energy theory, the mechanical test had also been utilized to study the adhesion performance. To date, a standard method to accurately determine the mechanical bond strength between asphalt and aggregate has still not been established. The existing researches in recent years mainly utilized the devices or methods in other industries, for example, pneumatic adhesion tensile testing instrument (PATTI) in the coatings and adhesive industry. The thin-film tension tests using PATTI were employed in pavement engineering to investigate the adhesion bond performance between aggregate and asphalt binder with water conditioning and dry conditions [10–14].

The adhesion bond performance was also presented to be more vital to the durability of porous asphalt mixes. With fewer fines in the mix, the bond between asphalt and aggregates is much more susceptible to traffic loading and environmental effects. To characterize the adhesion bond strength, the researchers from Delft University of Technology developed the thin-film adhesion test method and investigated the adhesion fatigue performance under traffic load [15, 16]. The frequency for this fatigue test was 10 Hz, which was usually used to characterize the effect of traffic load. However, as to the environmental effects such as moisture and freeze-thaw, a lower load frequency was expected to be more suitable.

In this paper, a low-frequency adhesion fatigue test was proposed to study the adhesion performance between aggregate and asphalt binder under the effect of environment. Different kinds of aggregates and asphalt binders (unaged, short and long term aged) were selected to analyze the factors influencing their adhesion fatigue performance.

## 2. Materials and Experimental Methods

### 2.1. Materials

**Figure 1:**
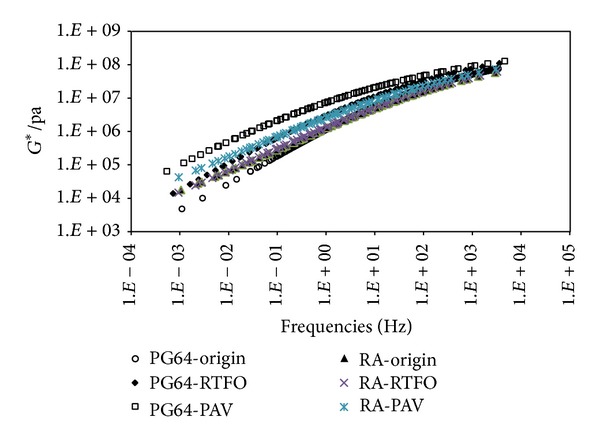
Master curves of G* for two kinds of asphalt at 20°C.

Two types of binders, PG64-22 and rubber asphalt (RA), were used in this study. Rolling thin-film oven test (RTFOT) and pressure aging vessel (PAV) test were utilized to simulate the short-term and long-term aging for asphalt binder. The shear dynamic moduli G* of the binders at different frequencies and temperatures were tested using conventional DSR and their master curves at 20°C were plotted in Figure 1. As shown in the figure, short-term and long-term aging have significant effects on the modulus of PG64-22, while less effect for aging is found on the modulus of rubber asphalt. When frequency is high enough, the effect of aging can be ignored for both of asphalt binders.

Granite and basalt were selected to be the test aggregates. The silicon dioxide contents are 47.95% for basalt and 66.43% for granite, respectively.

### 2.2. Sample Preparations

**Figure 2:**
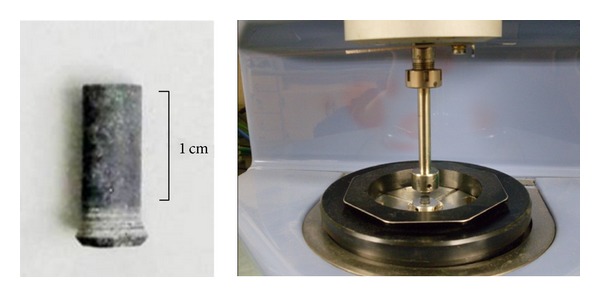
Column aggregate sample and setup of sample.

The column aggregate samples were prepared through cutting and coring the rock. The final column aggregate had a diameter of 5.72 mm and length of about 15 mm, as shown in Figure 2.

The adhesive bond mechanisms between aggregate and binder can be generally classified as mechanical interlocking and physical or chemical bonds. The physical and chemical bonds, as mentioned before, are generally classified as weak boundary layers, electrostatic forces, chemical bonding, and adhesion due to surface free energy. And the mechanical interlocking is determined by the aggregate surface morphology. This paper mainly focused on the physical and chemical bonds between aggregate and binder; thus the surface of aggregate samples was sanded and polished to keep the same surface texture.

The asphalt film thickness plays a critical role in investigating the adhesive bond performance between asphalt and aggregate. Many researchers had presented that the asphalt film thickness is about 6 to 10 microns for asphalt mixes with dense gradations, on the basis of calculating the asphalt content and aggregate surface area [17, 18]. As to the porous asphalt mixtures, the required asphalt film thickness was larger for the less aggregates and higher asphalt content. Therefore the Chinese specification recommends that the asphalt film thickness for porous asphalt mixes should be more than 14 microns. At last, 20 microns were selected to be the test film thickness for asphalt binder.

The conventional aluminum plates for DSR equipment were replaced with aggregate cylinder samples as shown in Figure 2. Thus a sandwich structure, which means two aggregate samples bonded with 20 *μ*m asphalt film, can be setup and utilized to investigate the adhesion fatigue performance between aggregate and asphalt.

### 2.3. Experiment Program

**Figure 3:**
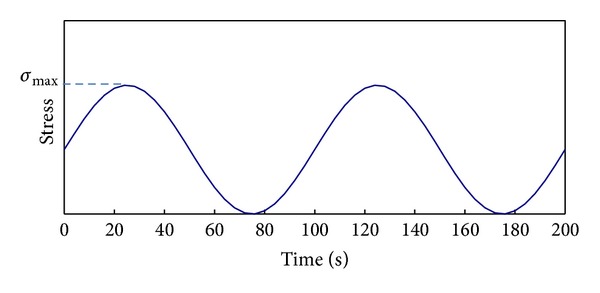
Stress-time curve for fatigue test.

The adhesion fatigue tests with stress-controlled mode were conducted using dynamic shear rheometer (DSR). The normal stress-time curve under fatigue load can be shown in Figure 3. In this paper, the environmental effects such as moisture and freeze-thaw had been studied. The effect of moisture can be realized by using the water bathing system of DSR. As to the frost expansion of water, it is clear that this effect varies along with the change of temperature. Therefore 0.01 Hz was finally selected to be the test frequency, which had been used to investigate the low-temperature fatigue performance of asphalt mixtures [19]. The experimental plan can be shown in Table 1.

In fatigue test, the definition of failure point is important. For each loading cycle, the modulus can be calculated through dividing peak stress by peak strain. In this paper, the 50% of initial modulus was finally defined as the failure of adhesion between asphalt and aggregate.

## 3. Results and Discussions

### 3.1. Fatigue Model

A fatigue damage model was firstly introduced to describe the relationship between fatigue life and stress amplitude. This model had been presented by Mo et al. and Huurman and Mo to study the fatigue performance for asphalt mastic and asphalt-aggregate adhesion under traffic load [15, 16]. In this model, the damage rate can be expressed as a function of applied stress amplitude:

(1) $$\begin{array}{c} \dot{D}={\left(\frac{\tau_{1}}{\tau_{0}}\right)}^{n}, \end{array}$$

where *τ*_1_ is applied shear stress amplitude (MPa); *D* is damage variable, for undamaged materials *D* = 0 and for complete rupture *D* = 1; *n* is model parameter; *τ*_0_ is reference shear stress (MPa).

It is also supposed that $\dot{D}=0$ when *τ*_1_ < 0. As indicated before, *D* = 0.5 is thought to be as the critical failure value in this paper.

For the cyclic loading, the damage increment can be indicated as follows:

(2) $$\begin{array}{c} \Delta D=D^{t+\Delta t}-D^{t}={\left(\frac{\tau_{1}^{t+\Delta t}+\tau_{1}^{t}}{2\tau_{0}}\right)}^{n}\times \Delta t, \end{array}$$

where *D* ^*t*+Δ*t*^ and *D* ^*t*^ are the cumulative damage at *t* + Δ*t* and *t*;  *τ*_1_^*t*+Δ*t*^ and *τ*_1_^*t*^ are shear stress at *t* + Δ*t* and *t* (MPa);  Δ*t* is time increment (s).

**Figure 4:**
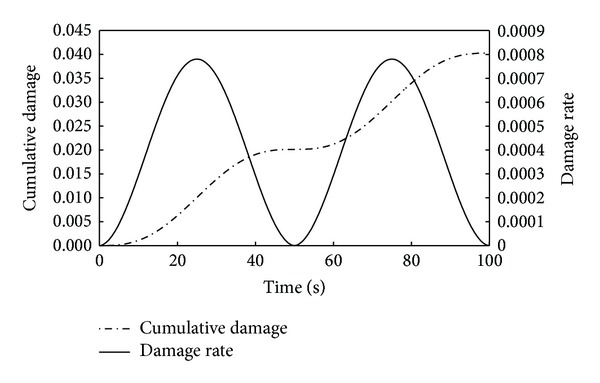
Damage rate and cumulative damage versus load time in a single loading cycle (20°C, rubber asphalt-PAV, stress amplitude 0.5 MPa)

At last, the cumulative damage in single loading cycle can be expressed as *D*_*s*_ = ∑Δ*D*. The damage rate and cumulative damage in single load cycle can be plotted in Figure 4. The same stress amplitude was supposed to induce the constant damage in every cycle. Therefore the fatigue life can be expressed as follows:

(3) $$\begin{array}{c} N_{f}=\frac{1}{2D_{s}}, \end{array}$$

where *D*_*s*_  is the damage caused by each loading cycle.

Because the asphalt is a typical viscoelastic material, the model parameters in (1) were also temperature dependent. Hence the linearity was supposed to describe the relationships between temperatures and model parameters, which can be also written as follows [16]:

(4) $$\begin{array}{c} \tau_{0}=a_{1}+b_{1}\cdot T\\ n=a_{2}+b_{2}\cdot T. \end{array}$$

Initial values for these parameters (*a*_1_, *a*_2_, *b*_1_, *b*_2_) in model were firstly set, and then the fatigue life could be calculated using (2) and (3). The error between the calculated fatigue lives and the experimental fatigue lives was calculated and minimized using least square error methods by adjusting the parameters iteratively. The optimized sets of final parameters of the equations were then obtained.

### 3.2. Validation of Fatigue Model

**Figure 5:**
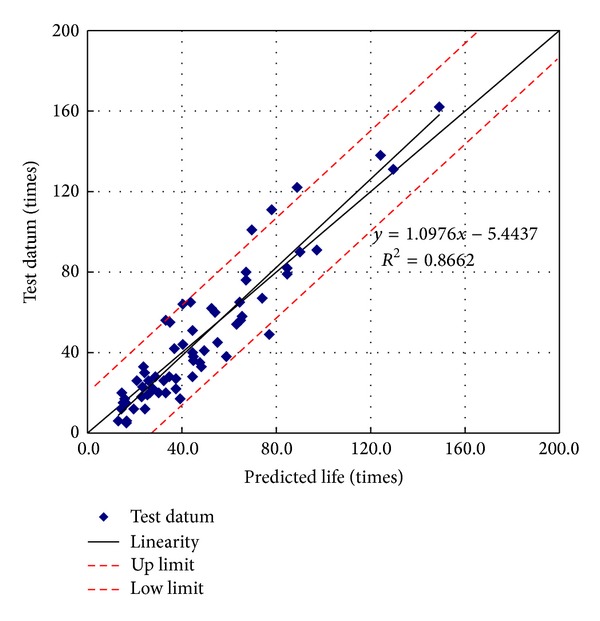
Experimental and predicted adhesion fatigue lives for basalt and different asphalt.

The fatigue lives for basalt and different asphalt were firstly investigated using the above fatigue model. The test data and predicted values are shown in Figure 5.

The two-sample *t*-tests and two-variance statistical tests were employed to validate the efficiency of fatigue model. The results can be found in Tables 2 and 3.

Tables 2 and 3 indicate that the fatigue model can predict the fatigue lives well. Similar studies had been conducted on the granite and different types of asphalt and same validation for the model could be proved.

### 3.3. Effect of Temperatures and Stress Amplitude

**Figure 6:**
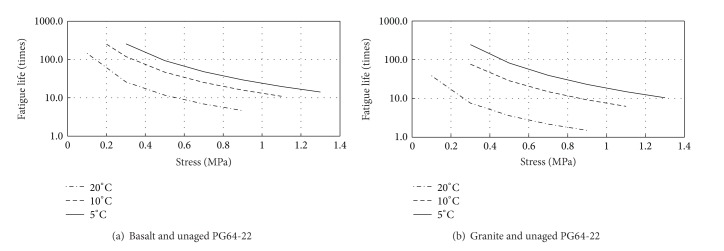
Predicted fatigue lives for aggregate and unaged asphalt (semilog).

The effect of temperature on the adhesion fatigue performance was firstly studied. The fatigue life curves in Figure 6 were obtained through determining the fatigue model parameters.

It is rather clear about the influence of temperatures and stress amplitude. The smaller applied stress amplitude and lower test temperature are able to increase the adhesion fatigue performance between aggregate and asphalt. Temperature is also found to have more significant effect on the granite-asphalt system. Due to the viscoelasticity of asphalt material, the decrease of temperature induces to the increase of asphalt's stiffness and adhesion to aggregate. However, if temperature continues to reduce to be lower than brittle point of asphalt, the adhesion performance between asphalt and aggregate is supposed to deteriorate rapidly.

### 3.4. Effect of Asphalt Binder and Aging

**Figure 7:**
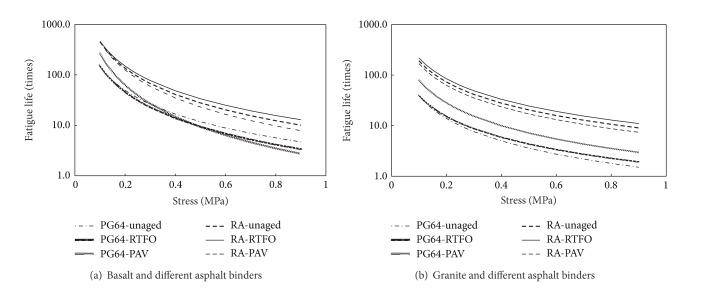
Predicted fatigue lives (semilog) for aggregate and different asphalt binders.

The predicted adhesion fatigue lives can be shown in Figure 7 for basalt/granite and different types of asphalt binders.

Generally, rubber asphalt has much better adhesion fatigue performance to aggregate, when compared with PG64-22 binder. However, the effect of binder's aging on the adhesion fatigue performance is different for PG64-22 and rubber asphalt. Aging, especially long-term aging (PAV), can improve the adhesion fatigue performance between granite aggregate and PG64-22 binder. This means that aging to PG64-22 binder can be helpful to bond with granite aggregate and resist the influence of environmental effects. Although the basalt and PG64-22 system with PAV and RTFOT aging has lower fatigue lives at high stress amplitude, as shown in Figure 7(a), all the absolute fatigue lives are very small (less than 10 times). Therefore, a small property difference among aggregate samples can result in the variation of fatigue lives in the high stress amplitude scope.

Aging is found to play a less role in the adhesion fatigue performance between aggregate and rubber asphalt. Specifically RTFOT aging can help to improve the adhesion performance, while the effect of PAV aging is negative. Thus it can be concluded that increasing stiffness of asphalt at a certain extent can be helpful to its adhesion to aggregate. But instead excessive increase to asphalt's stiffness weakens their bond between asphalt and aggregate.

### 3.5. Effect of Aggregate Property

**Figure 8:**
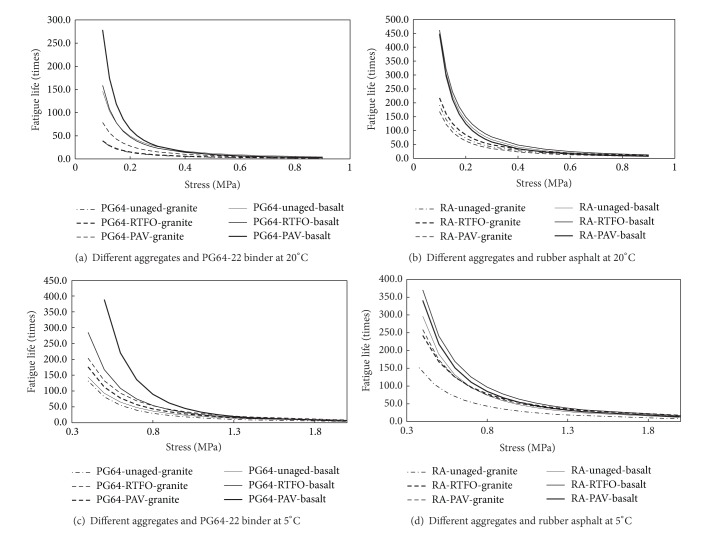
Fatigue lives for different aggregates and asphalt binders at 5°C and 20°C.

To investigate the effect of aggregate property, the fatigue lives at different stress amplitudes and temperatures (20°C and 5°C) are plotted in Figure 8.

If classified by the content of SiO_2_, the basalt is regarded as hydrophobic aggregates with low silica content, while granite is considered to be the hydrophilic aggregates, which generally has a high content of silica [20]. Generally, basalt is commonly used in pavement construction around the world. But granite is also used in many roads for its high resistance to abrasion. Although many researchers presented that granite had a bad physiochemical bond to asphalt, someone also pointed that the high surface textures or holes for granite lead to the good bond between granite and asphalt [9].

Figure 8 demonstrates the better adhesion performance existing between basalt and asphalt if compared with granite. However, this difference is reduced along with the increase of applied stress amplitude. The effect of aggregate is more significant to unmodified asphalt (PG64-22) when compared with rubber asphalt.

**Figure 9:**
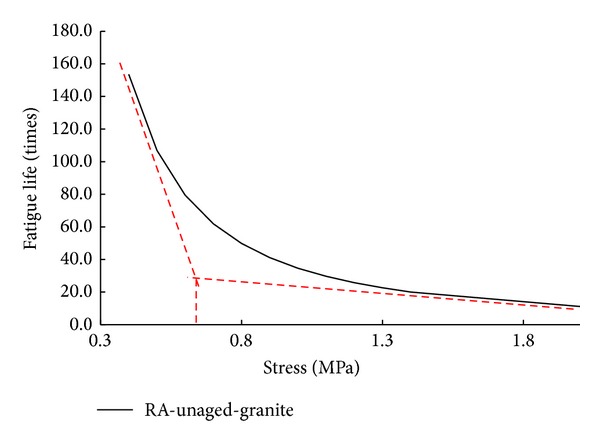
Schematic view for critical stress amplitude of fatigue curve.

For all the curves of fatigue lives in Figures 6 to 8, there is an obvious inflection point, which indicates the change of damage rate caused by applied stress, as shown in Figure 9. This point can be defined as critical stress amplitude. Specifically the stress is around 0.2-0.3 MPa for test datum at 20°C, and 0.6-0.7 MPa for datum at 5°C. After calculating the real load caused by environmental factors in asphalt mixture with field monitoring or theoretical calculation (FEM, etc.), the critical stress amplitude could be used to estimate the possibility of premature damage in asphalt pavement.

To sum up from Figures 6 to 9, the aggregate property plays a more important role in the adhesion fatigue performance, especially when bonded asphalt is unmodified binder (PG64-22). As to the modified asphalt (rubber asphalt), the adhesion performance between asphalt and aggregate is more dependent on the asphalt property.

## 4. Conclusions

The adhesion experimental method for thin-film asphalt and aggregate system was firstly developed to investigate the effect of environmental factors in this paper. Then the factors influencing the adhesion performance between asphalt and aggregate were studied.

The used fatigue model can successfully predict the adhesion fatigue lives for different types of asphalt and aggregates. When applied stress amplitude and test temperature increase, the adhesive fatigue lives between asphalt and aggregate deteriorate rapidly.

Modified asphalt (rubber asphalt) has better aging-resistance and stronger adhesion to aggregate comparing with unmodified asphalt (PG64-22). The RTFOT and PAV aging to PG64-22 can improve its adhesion to aggregate. However, the function of long-term aging is different for rubber asphalt. It is found that the long-term aging has negative effect on the adhesive fatigue performance between aggregates and rubber asphalt.

Basalt, which is regarded as hydrophobic aggregate, has better adhesive fatigue performance to asphalt binder compared with granite. However, the effect of aggregate property is reduced if applied stress amplitude increases. To sum up, the influence of asphalt property is more dominant to the adhesion fatigue performance between modified asphalt and aggregate. If used binder in asphalt mixture is unmodified asphalt, the effect of aggregate property cannot be ignored.

## Figures and Tables

**Table 1:** Experimental plan for adhesion fatigue test.

Aggregate	Asphalt	Test temperature/°C	Stress amplitude/MPa
Basalt	PG64-22 unaged, RTFO, PAV; RA unaged, RTFO, PAV	5	0.6, 0.8, 1.0, 1.2
		10	0.6, 0.8, 1.0
		20	0.2, 0.3, 0.4, 0.5

Granite	PG64-22 unaged, RTFO, PAV; RA unaged, RTFO, PAV	5	0.8, 1.0, 1.2
		10	0.6, 0.8, 1.0
		20	0.2, 0.3, 0.4

**Table 2:** *t*-test for experimental and predicted adhesion fatigue lives.

Null hypothesis (*H*_0_)	Confidence level	Sample size	Average difference	Standard deviation of difference	*P* value	Conclusion
Difference = 0	95%	63	−0.767	12.988	0.641	Accept *H*_0_

**Table 3:** Variance test for experimental and predicted adhesion fatigue lives.

Null hypothesis (*H*_0_)	Confidence level	Sample size	Degree of freedom	Statistics for *F*-test	*P*value	Conclusion
Equal variance	95%	63	62	1.391	0.197	Accept *H*_0_
